# Do patients and carers agree on symptom burden in advanced COPD?

**DOI:** 10.2147/COPD.S147892

**Published:** 2018-03-22

**Authors:** Emma Mi, Ella Mi, Gail Ewing, Patrick White, Ravi Mahadeva, A Carole Gardener, Morag Farquhar

**Affiliations:** 1 School of Clinical Medicine; 2 Centre for Family Research, University of Cambridge, Cambridge; 3 Primary Care and Public Health Sciences, King’s College London, London; 4 Department of Respiratory Medicine, Cambridge NIHR BRC, Addenbrookes Hospital; 5 Department of Public Health and Primary Care, University of Cambridge, Cambridge; 6 School of Health Sciences, University of East Anglia, Norwich, UK

**Keywords:** COPD, informal carers, symptom, agreement

## Abstract

**Objective:**

Accurate informal carer assessment of patient symptoms is likely to be valuable for decision making in managing the high symptom burden of COPD in the home setting. Few studies have investigated agreement between patients and carers in COPD. We aimed to assess agreement between patients and carers on symptoms, and factors associated with disagreement in a population-based sample of patients with advanced COPD.

**Patients and methods:**

This was a prospective, cross-sectional analysis of data from 119 advanced COPD patients and their carers. Patients and carers separately rated symptoms on a 4-point scale. Wilcoxon signed-rank tests and weighted Cohen’s kappa determined differences in patient and carer scores and patient–carer agreement, respectively. We identified characteristics associated with incongruence using Spearman’s rank correlation and Mann–Whitney *U* tests.

**Results:**

There were no significant differences between group-level patient and carer scores for any symptom. Patient–carer individual-level agreement was moderate for constipation (k=0.423), just below moderate for diarrhea (k=0.393) and fair for depression (k=0.341), fatigue (k=0.294), anxiety (k=0.289) and breathlessness (k=0.210). Estimation of greater patient symptom burden by carers relative to patients themselves was associated with non-spousal patient–carer relationship, non-cohabitating patients and carers, carer symptoms of anxiety and depression and more carer unmet support needs. Greater symptom burden estimation by the patient relative to the carer was associated with younger patients and longer duration of COPD.

**Conclusion:**

Overall, agreement between patients and carers was fair to moderate and was poorer for more subjective symptoms. There is a need to encourage open dialogue between patients and carers to promote shared understanding, help patients express themselves and encourage carers to draw attention to symptoms that patients do not report. The findings suggest a need to screen for and address both the psychological morbidities in patients with advanced COPD and their carers and unmet support needs in carers.

## Plain language summary

Patients with COPD are affected by many symptoms such as breathlessness and tiredness in their day-to-day life. Family and friends (informal carers) often have to help manage these symptoms and decide when to get medical help. We wanted to find out whether patients and their carers agree in their judgments of the patient’s symptoms and what factors are associated with better or worse agreement as this may influence how well symptoms are managed, given the carer’s role in this. One hundred and nineteen patients and their carers separately rated how much the patient was bothered by six common symptoms in COPD. Agreement between patients and carers was worse for less obvious symptoms such as depression, fatigue, anxiety and breathlessness than constipation and diarrhea. Carers who said that their patient had more problematic symptoms than the patients themselves tended to have a non-spousal relationship with and live apart from their patient, be symptomatically anxious or depressed themselves and have more unmet support needs. Patients reporting a greater symptom burden than their carer reported for them were younger and had COPD for longer. Our results suggest clinicians should seek opinions about symptoms from both the patient and their carer, promote shared understanding between patients and carers, and screen for mental health problems in both and unmet support needs in carers.

## Introduction

COPD carries a high symptom burden, including psychological impacts such as anxiety and depression. Accurate symptom assessment by informal carers, typically family and friends, is likely to be important for their own decision making in supporting symptom management in the home setting and in deciding when to seek medical help.^6^,^7^ Inaccurate assessment may negatively impact on the quality of the patient–carer relationship and the psychological health of both, and may lead to overtreatment or inadequate symptom control.^1^,^6^,^8^–^10^

The extent of agreement between patients and carers is debated. There is widespread consensus in cancer that, at the group patient and carer cohort level, carers consistently overestimate patient symptoms,^2^–^6^,^8^,^9^,^11^–^16^ although the magnitude of the bias is small.^2^,^11^,^13^,^17^ At the individual patient–carer dyad level, strength of agreement on symptoms ranges from fair to substantial.^2^,^4^,^11^,^12^,^14^–^19^ It is well established in cancer populations that there is greater agreement between patients and carers for more observable symptoms (typically physical symptoms) compared to less observable symptoms (typically psychological symptoms).^3^–^5^,^8^,^13^,^15^ Group-level carer bias toward over-reporting is small for physical symptoms but moderate for psychological symptoms, with overestimation primarily for fatigue, pain, anxiety and depression.^1^,^5^,^11^,^13^ Individual-level agreement is substantial on physical symptoms compared to only fair on psychological symptoms, with substantial agreement in nausea and vomiting, moderate agreement in pain, poor sleep, poor appetite and constipation, and poor to fair agreement in fatigue, anxiety and depression.^2^,^3^,^11^–^13^,^15^,^18^

Previous studies have investigated patient and carer characteristics associated with symptom disagreement in cancer, with inconsistent links to carer age,^3^,^9^,^10^,^12^,^18^,^20^,^21^ sex,^3^,^10^,^12^,^13^,^21^ patient–carer relationship^3^–^5^,^11^,^20^ and cohabitation,^22^ moderate disease severity,^1^,^21^ poor patient and carer psychological health,^1^,^13^,^20^,^23^,^24^ greater subjective caring burden,^1^,^3^,^8^–^10^,^20^,^23^ lack of carer support and low carer self-esteem.^3^,^12^

Agreement between patients and carers has been extensively investigated in cancer (including lung cancer),^6^,^12^ but few studies have examined this in COPD^19^ or in population-based samples. We assessed agreement on how bothered the patient was by symptoms of breathlessness, fatigue, constipation, diarrhea, anxiety and depression. We took a holistic approach in considering the experience of those living with advanced COPD; thus, we included a spectrum of physical and psychological symptoms resulting from the condition, side effects of treatments, symptoms of conditions commonly comorbid with advanced COPD and symptoms which are difficult to manage in advanced COPD as the action of managing them leads to breathlessness, for example, walking to and using the toilet. We aimed to determine agreement between patients and carers on these symptoms, and factors associated with disagreement in a population-based sample of patients with advanced COPD.

## Patients and methods

### Study design and participants

We conducted a cross-sectional analysis of data from patients and carers participating in baseline interviews for the Longitudinal Interview Study component of the prospective Living with Breathlessness Study.^25^ Primary care patients with well-characterized advanced COPD were recruited, if they met at least two of six inclusion criteria: forced expiratory volume in 1 second <30% predicted, at least two exacerbations requiring prednisolone or antibiotics in the previous year, at least one hospital admission for COPD in the previous 2 years, long-term oxygen therapy, cor pulmonale and Medical Research Council Dyspnea scale 4+. Patients were excluded if they had a serious mental health problem, serious learning difficulty, active cancer or active alcoholism. Patients were asked to identify their primary informal carer (defined as the person who provided the most help and support and was not a health care professional) and these carers were also invited to participate. Carer exclusion criteria were as follows: under 18 years of age and unable to give informed consent. Care provided by carers could encompass personal care, for example, walking, dressing, washing, toileting as well as help with practical tasks, including shopping, cooking, housework, transport and paperwork. Six hundred and fifty-two patients with advanced COPD were identified by 63 primary care practices in the East of England (targeted to maximize variability and representativeness), and were mailed a recruitment pack. Two hundred and thirty-five patients consented and participated (36% participation rate), from whom 117 carers were recruited to the study (50% participation rate; not all patients identified a carer). The study received ethics approval from the National Research Ethics Service Committee East of England – Cambridge South (reference number 12/EE/0163). All participants gave written informed consent.

### Data collection

Data were collected by conducting separate face-to-face interviews with the patients and carers. Sociodemographics of participants and measures of patients’ disease severity (% predicted forced expiratory volume in 1 second, Medical Research Council dyspnea scale, COPD Assessment Test score, number of hospital admissions, number of exacerbations at home, long-term oxygen use) were obtained.^26^,^27^ Psychological morbidity was identified for both patients and carers using the 14-item (two 7-item subscales) Hospital Anxiety and Depression Scale (HADS), with caseness for anxiety and depression defined as HADS-A and HADS-D score ≥11 (on a scale of 0–21).^28^ HADS was used as it is a well-validated and reliable self-report screening tool encompassing symptoms of both anxiety and depression, and has been shown to have reliability, convergent/discriminant validity and responsiveness to change in the primary care population.^29^ Carers identified unmet support needs on the 14-item Carer Support Needs Assessment Tool (CSNAT). CSNAT covers 14 broad support domains in two groupings: support to enable the carer to provide care for the patient (these “enabling” domains include understanding the illness, knowing what to expect in the future, knowing who to con-tact if concerned, talking with the patient about the illness, managing symptoms, providing personal care, equipment to help care) and support for carers’ own health and well-being (these “direct” domains include looking after own physical health, dealing with feelings and worries, support with beliefs or spiritual concerns, support with financial, legal and work issues, practical help at home, having time to oneself in the day, having a break from caring overnight). The need for more support is rated on a 4-point scale from 0 (none) to 3 (very much more). There is an “anything else” section enabling the carer to write in any other support need they may have that is not covered by the other 14 items.^30^ The CSNAT tool has been validated in a multicenter UK cohort of carers of palliative patients, demonstrating good face, content and criterion validity, and is being used in populations in Australia, USA, Canada, Europe and China.^31^ The CSNAT was used as it is the only validated screening tool which directly measures carers’ support needs (as opposed to the multiple tools that measure carer burden or distress, which are only indirect indicators of need). Patients and carers rated patient symptoms of breathlessness, fatigue, constipation, diarrhea, anxiety and depression (patients and carers were asked: “how much were you/was the patient bothered by the symptom in the past week”) on 4-point scales: 1 (not at all), 2 (a little), 3 (quite a bit) and 4 (very much).^32^

### Statistical analysis

Wilcoxon signed-rank test was used to determine whether differences between patient and carer scores were significant at the group level. The direction and magnitude of mean differences (mean scores of carers minus patients) were standardized to Cohen’s d effect size (statistical magnitudes of the observed bias), which were interpreted as 0.2= small, 0.5= moderate and 0.8= large.^33^ Agreement on symptoms between patients and carers on the individual level was evaluated by weighted Cohen’s kappa, with 0.81–0.99 defined as excellent agreement, 0.61–0.80 as substantial agreement, 0.41–0.60 as moderate agreement, 0.21–0.40 as fair agreement and ≤0.20 as poor agreement.^34^ Spearman’s correlation coefficients were used to identify the strength of association between difference in symptom burden estimates by patients and carers (carer score minus patient score) and variables of interest; positive correlation represents greater carer estimation relative to the patient as the variable of interest increases. Mann–Whitney *U* tests identified categorical variables significantly associated with difference between patient and carer scores. Where variables were significant in univariate analysis, ordinal logistic regression was performed to determine significance when adjusted for patient and carer age and sex. Statistical significance was set at *p*≤0.05. All statistical analyses were carried out in SPSS Version 23 (IBM Corp., Armonk, NY, USA).

## Results

Mean patient and carer age was 71.39±8.69 and 64.16±14.50 years, respectively. Also, 62.4% of patients and 27.4% of carers were male; 80.9% of patients and carers were spouses and 87.2% lived together. Patient and carer characteristics are shown in Table 1.

Mean group-level differences and individual dyad-level agreement between patient and carer scores for all symptoms are shown in Table 2.

The most bothersome symptoms were breathlessness and fatigue, followed by anxiety and depression. There was no significant difference between mean patient and carer scores for any symptom at the group level. Agreement between patients and carers on symptoms at the individual dyad level was moderate for constipation, just below moderate for diarrhea and fair for depression, fatigue, anxiety and breathlessness.

Factors associated with disparity in dyad-level patient and carer scores are shown in Table 3.

Carers being more likely to estimate greater symptom burden in patients relative to patients themselves was associated with non-spousal patient–carer relationships, non-cohabitation of patients and carers, carer caseness for anxiety and depression on HADS and unmet support needs in more CSNAT domains, specifically more direct carer support domains. Greater symptom burden estimation by the patient relative to the carer was associated with younger patients and longer self-reported duration of COPD. However, the strength of correlation between patient–carer disparity and carer unmet support needs, age and duration of COPD was weak. In multivariate analysis (Table 4), the association between unmet support needs in more direct carer support domains and greater estimation of breathlessness by carers remained when adjusted for patient and carer age and sex (odds ratio 1.250, 95% CI 1.031–1.516), as did younger patient age and greater patient estimation of depression (odds ratio 1.090, 95% CI 1.018–1.167).

## Discussion

Unlike previous studies in cancer which have found a bias toward carer overestimation, there was no significant difference in this population-based sample of carers and patients with advanced COPD between their ratings of any symptom at the group level. However, agreement between patients and carers at the individual dyad level did not exceed moderate for any symptom, indicating lack of agreement between patients and carers within individual dyads which are masked at the group (cohort) level. What is not known is whether the patient’s or the carer’s reporting reflects the “true” symptom experience, and previous studies have suggested that the patient’s view cannot be considered to be the gold standard. However, it could be that there is no “true” experience and that alternative viewpoints are valuable.^35^

There are several possible explanations for disagreement. First, it is known that patients engage in protective buffering, concealing symptoms in order to minimize the burden on their carer.^12^,^21^,^24^ Response shift may occur in patients, with those with advanced disease reporting less symptom burden possibly because patients who are living with advanced COPD simply accept and consciously do their best to minimize their symptoms or subconsciously expect, and therefore experience, less symptom burden.^36^,^37^ Carers may find it less easy to accept symptoms, and their impacts, leading to estimation of greater symptom burden by the carer. Conversely, limited carer presence at patient–clinician contacts may result in lower carer awareness of symptoms and, therefore, lower estimation of symptom burden. Interestingly, there was a tendency toward lower carer estimation for five of the six symptoms, possibly reflecting longer disease trajectories in COPD and slower disease progression, compared to previous studies in cancer, leading to carer compassion-fatigue and response shift.

We found higher agreement for more observable physical symptoms such as constipation and diarrhea than less observable psychological symptoms such as anxiety and depression and those with emotional valence such as fatigue and breathlessness. This can be attributed to greater difficulty appraising psychological symptoms due to a lack of overt and non-verbal cues and, therefore, the need for greater interpretation of cues, which carers may not have the knowledge or skills to do,^3^,^5^,^12^ but which could be gained. It may also be easier for patients to conceal psychological symptoms, and carers may be more prone in these circumstances to make assessments colored by their individual frame of reference.^6^,^12^,^24^ In addition, constipation and diarrhea were less-frequent and episodic symptoms, which may have increased carers’ awareness of them as a notable change in contrast to the more daily-present symptoms such as breathlessness and fatigue.

Estimation of greater patient symptoms of anxiety and depression by carers was seen in non-spousal and non-cohabitating patient–carer dyads, probably because these carers have less face-to-face contact with their patients,^5^,^22^ so have less opportunity to observe particularly subjective symptoms. Carer anxiety and depression caseness on the HADS scale were also associated with estimation of greater patient symptoms of anxiety and depression by carers, probably due to projection of their own experiences and feelings, thus creating a more negative view of the situation.^1^,^12^ Higher carer rating of breathlessness was associated with unmet carer support needs across a wider range of carer support domains (number of CSNAT domains) and interestingly only across more domains of direct support for carer health and well-being rather than support to enable the carer to provide care for the patient, which persisted when adjusted for patient and carer age and sex; this may suggest that carer perception of the patient’s breathlessness is related to the carer’s own physical, psychological and spiritual well-being, more than difficulties in managing it.^38^ Estimation of greater depressive symptoms by relatively younger patients found in univariate and multivariate analyses may reflect the greater difficulty of psychologically adjusting to disease limitations at an earlier stage in life, although given that the cohort was, in general, relatively old (reflecting being population based with advanced COPD), even those who were “younger” may not be “young” in the broader COPD population.

These findings have implications for clinical practice and research. Given the only moderate agreement between patients and carers, clinicians and researchers should, in the context of COPD, seek the perceptions of both.^35^ Clinicians may be well placed to open a dialogue between patients and carers within dyads,^8^ helping them to gain a shared understanding of each other’s position and address any differences in perceptions. One approach that has been found to be of benefit is perspective taking, which involves imagining the patient’s perspective rather than assuming their feelings (the cognitive, rather than the affective, dimension of empathy).^39^ In addition, we need to find ways of helping patients express themselves and ensure that we respond to their needs without forcing them to acknowledge or experience their illness more than they want to. Carers can have a role in this, helping to draw attention to symptoms that patients do not emphasize and considering whether patients need help in being a little more demanding about some of their needs. To help promote agreement, screening for and addressing psychological morbidities in both patients and carers and unmet support needs in carers is required. However, patient assessment for anxiety and depression is itself not widespread in respiratory clinics (or indeed for any chronic disease and among all health professionals), so it may be challenging to implement carer assessment of these in a clinical setting. Finally, further investigation of the impact of patient–carer disagreement is warranted.

## Limitations

One of the limitations of the study is that the cohort was limited to patients with advanced COPD and, therefore, may not represent the majority of those with COPD. Nevertheless, disease severity was not found in this study to be associated with agreement between patients and carers, so it is possible that similar patient–carer agreement relationships may be found in general COPD populations. Additionally, data were not available for other symptoms associated with COPD, such as cough. We used the HADS screening tool to identify caseness for anxiety and depression in patients and carers, which is not a formal diagnosis of anxiety or depression, but the stringent definition of caseness of HADS score ≥11 (moderate to severe depression) may have improved accuracy. Finally, the cross-sectional analysis could establish association, but not causality.

## Conclusion

Agreement between patients and carers was fair to moderate and was poorer for less-observable symptoms. Greater symptom burden estimation by the carer was associated with less patient–carer contact, carer symptoms of anxiety and depression and a wider range of unmet carer support needs; greater estimation by the patient was associated with being younger and having COPD for longer. There is a need to encourage open dialogue between patients and carers to promote shared understanding of patients’ symptom burden. This may encourage carers to explore the impact of symptoms that patients do not emphasize. Screening for and addressing psychological morbidities in patients with advanced COPD and their carers, and identifying and responding to unmet support needs in carers may identify opportunities for supportive intervention. There is a potential to develop a useful therapeutic dialogue addressing the complimentary viewpoints of patients and carers, and clinicians.

## Figures and Tables

**Table 1:** Sociodemographic and clinical characteristics of patients and carers

Characteristics	Patients	Carers
Sample size	117	117
Age, years	71.39 (8.69)	64.16 (14.50)
Sex, male, n (%)	73 (62.4)	32 (27.4)
Marital status, n (%)		
Single	1 (0.9)	7 (6.1)
Partner	99 (85.3)	97 (85.1)
Separated/divorced/widowed	16 (13.8)	10 (8.8)
Living alone, n (%)	12 (10.3)	3 (2.7)
Educational level, n (%)		
None	54 (47.8)	53 (47.3)
A level/O level	18 (15.9)	17 (15.2)
Higher	18 (15.9)	19 (17.0)
Others	23 (20.4)	23 (20.5)
Occupation, n (%)		
Higher managerial/professional	6 (5.2)	4 (3.5)
Lower managerial/professional	10 (8.6)	7 (6.2)
Non-manual	40 (34.5)	57 (50.4)
Skilled manual	5 (4.3)	11 (9.7)
Semi-skilled manual	35 (30.2)	13 (11.5)
Unskilled	20 (17.2)	21 (18.6)
Income, n (%)		
<£220/week	20 (19.0)	17 (17.0)
£221–£350/week	42 (40.0)	39 (39.0)
£351–£505/week	19 (18.1)	19 (19.0)
£506–£732/week	13 (12.4)	15 (15.0)
>£733/week	11 (10.5)	10 (10.0)
Patient–carer relationship, n (%)		
Spouse	93 (80.9)	
Child	14 (12.1)	
Other	8 (7.0)	
Patient–carer cohabitation, n (%)	102 (87.2)	
Smoking, n (%)		
Current	20 (17.2)	
Former	93 (80.2)	
Never	3 (2.6)	
Duration of COPD, years	11.36 (10.49)	
% predicted FEV_1_	38.23 (20.41)	
MRC dyspnea scale score	3.64 (1.06)	
CAT score	23.43 (7.16)	
No of hospital admissions	0.53 (0.86)	
No of exacerbations at home	2.94 (3.46)	
Long-term oxygen therapy, n (%)	22 (20.2)	
Duration of caring, years		11.34 (13.52)
Hours of care, n (%)		
<19 h/week		42 (39.3)
20–49 h/week		29 (27.1)
>50 h/week		36 (33.6)
Patient comorbidities	3.91 (2.00)	
Carer comorbidities		1.47 (1.48)

**Note:** All variables are expressed as mean (SD) unless otherwise stated.

**Abbreviations:** CAT, COPD assessment test; FEV_1_, forced expiratory volume in 1 second; MRC, Medical Research Council.

**Table 2:** Mean group-level differences and individual dyad-level agreement between patient and carer symptom ratings

Symptoms	Sample size	Patient mean score (SD)	Carer mean score (SD)	Absolute mean difference (SD)	Standardized mean difference	Wilcoxon signed-rank test *p*-value^a^	Weighted Cohen’s kappa (95% CI)^b^
Breathlessness	106	3.25 (0.68)	3.10 (0.85)	−0.151 (0.934)	−0.162	0.089	0.210 (0.053–0.367)
Fatigue	105	2.90 (0.99)	2.84 (0.91)	−0.067 (1.103)	−0.061	0.571	0.294 (0.161–0.427)
Anxiety	96	2.22 (1.08)	2.20 (1.05)	−0.021 (1.196)	−0.018	0.749	0.289 (0.137–0.440)
Depression	100	1.90 (1.06)	1.81 (0.94)	−0.090 (1.055)	−0.085	0.342	0.341 (0.195–0.486)
Constipation	91	1.60 (0.93)	1.58 (0.96)	−0.022 (0.954)	−0.023	0.840	0.423 (0.246–0.599)
Diarrhea	93	1.31 (0.59)	1.40 (0.75)	0.086 (0.702)	0.123	0.264	0.393 (0.231–0.555)

**Notes:**

a Mean group-level difference.

b Individual dyad-level agreement.

**Table 3:** Factors associated with disparity between individual dyad-level patient and carer symptom ratings

Factors	Breathlessness	Fatigue	Constipation	Diarrhea	Anxiety	Depression
Patient age, ρ (*p*-value)	0.009 (0.930)	0.073 (0.457)	0.050 (0.635)	0.086 (0.414)	−0.003 (0.977)	0.218 (0.029)*
Carer age, ρ (*p*-value)	−0.042 (0.672)	0.046 (0.646)	0.022 (0.836)	−0.100 (0.344)	−0.086 (0.412)	−0.143 (0.164)
Patient sex, mean difference^a^						
Male	−0.227	−0.123	0.034	0.000	−0.068	−0.097
Female	−0.025	0.025	−0.125	0.250	0.054	−0.079
*p*-value	0.304	0.630	0.600	0.224	0.770	0.908
Carer sex, mean difference^a^						
Male	−0.100	−0.035	−0.269	0.231	−0.179	−0.172
Female	−0.171	−0.079	0.077	0.030	0.044	−0.056
*p*-value	0.800	0.752	0.266	0.557	0.462	0.595
Patient–carer relationship, mean difference^a^						
Spouse	−0.151	−0.105	−0.066	0.104	−0.143	−0.198
Non-spouse	−0.150	0.105	0.200	0.000	0.474	0.368
*p*-value	0.900	0.610	0.666	0.692	0.048*	0.043*
Patient–carer cohabitation, mean difference^a^						
Yes	−0.151	−0.152	−0.085	0.108	−0.167	−0.193
No	−0.154	0.539	0.556	−0.100	1.000	0.667
*p*-value	0.653	0.063	0.150	0.478	0.002*	0.007*
Duration of COPD, ρ (*p*-value)	−0.052 (0.602)	−0.207 (0.035)*	−0.017 (0.870)	0.064 (0.542)	0.059 (0.570)	−0.107 (0.292)
% predicted FEV_1_, ρ (*p*-value)	−0.013 (0.934)	−0.078 (0.605)	0.029 (0.860)	0.050 (0.760)	0.013 (0.936)	0.134 (0.386)
Patient HADS-A caseness, mean difference^a^						
Yes	−0.226	−0.267	0.148	0.000	−0.226	−0.258
No	−0.110	−0.028	−0.129	0.129	0.065	−0.015
*p*-value	0.700	0.450	0.259	0.187	0.350	0.221
Patient HADS-D caseness, mean difference^a^						
Yes	−0.412	0.059	0.357	−0.071	0.000	−0.188
No	−0.092	−0.129	−0.120	0.118	−0.039	−0.074
*p*-value	0.348	0.536	0.165	0.343	0.749	0.621
Carer HADS-A caseness, mean difference^a^						
Yes	−0.069	0.000	0.208	0.039	0.520	0.400
No	−0.160	−0.093	−0.121	0.106	−0.229	−0.260
*p*-value	0.473	0.484	0.089	0.867	0.012*	0.017*
Carer HADS-D caseness, mean difference^a^						
Yes	−0.250	0.000	0.222	0.100	−0.200	0.800
No	−0.120	−0.076	−0.062	0.085	−0.012	−0.193
*p*-value	0.671	0.866	0.636	0.859	0.668	0.008*
No. of CSNAT domains, ρ (*p*-value)	0.246 (0.016)*	0.053 (0.610)	−0.152 (0.171)	0.179 (0.101)	0.220 (0.039)*	0.187 (0.075)
No. of direct CSNAT domains, ρ (*p*-value)	0.209 (0.035)*	0.054 (0.588)	−0.085 (0.430)	0.135 (0.205)	0.213 (0.041)*	0.167 (0.103)
No. of enabling CSNAT domains, ρ (*p*-value)	0.186 (0.066)	0.030 (0.771)	−0.194 (0.075)	0.195 (0.070)	0.165 (0.120)	0.134 (0.199)

**Notes:**

a Mann–Whitney *U* test was used for these variables. Spearman’s correlation was used for all other variables.

* *p*≤0.05.

**Abbreviations:** CSNAT, Carer Support Needs Assessment Tool; FEV_1_, forced expiratory volume in 1 second; HADS, Hospital Anxiety and Depression Scale.

**Table 4:** Multivariate analysis of factors associated with disparity between individual dyad-level patient and carer symptom ratings

Factors	Breathlessness, OR (95% CI)	*p*-value	Fatigue, OR (95% CI)	*p*-value	Anxiety, OR (95% CI)	*p*-value	Depression, OR (95% CI)	*p*-value
Patient age	1.003 (0.953–1.056)	0.911	1.087 (0.976–1.209)	0.128	0.934 (0.872–1.001)	0.055	1.090 (1.018–1.167)	0.014*
Carer age	1.002 (0.971–1.034)	0.890	0.920 (0.843–1.005)	0.064	1.065 (1.007–1.126)	0.027*	0.960 (0.910–1.014)	0.142
Patient sex	0.471 (0.140–1.586)	0.224	3.418 (0.325–35.993)	0.306	0.627 (0.153–2.579)	0.518	2.132 (0.503–9.029)	0.304
Carer sex	0.779 (0.215–2.822)	0.704	18.933 (0.800–448.281)	0.069	0.806 (0.196–3.322)	0.766	2.288 (0.536–9.773)	0.264
Patient–carer relationship					5.505 (0.528–57.353)	0.154	0.576 (0.058–5.731)	0.638
Patient–carer cohabitation					0.317 (0.060–1.678)	0.177	0.237 (0.042–1.324)	0.101
Duration of COPD			0.956 (0.873–1.046)	0.325				
% predicted FEV_1_			0.981 (0.952–1.011)	0.206				
Carer HADS-A caseness					0.435 (0.162–1.167)	0.098	0.507 (0.173–1.486)	0.216
Carer HADS-D caseness							0.244 (0.056–1.067)	0.061
No of direct CSNAT domains	1.250 (1.031–1.516)	0.023*			1.118 (0.896–1.396)	0.323		

**Note:**

* *p*≤0.05.

**Abbreviations:** CSNAT, Carer Support Needs Assessment Tool; FEV_1_, forced expiratory volume in 1 second; HADS, Hospital Anxiety and Depression Scale; OR, odds ratio.
